# The antiproliferative effect of *Moringa oleifera* crude aqueous leaf extract on cancerous human alveolar epithelial cells

**DOI:** 10.1186/1472-6882-13-226

**Published:** 2013-09-16

**Authors:** Charlette Tiloke, Alisa Phulukdaree, Anil A Chuturgoon

**Affiliations:** 1 Discipline of Medical Biochemistry, School of Laboratory Medicine and Medical Sciences, College of Health Sciences, University of KwaZulu-Natal, Durban South Africa; 2 Postal address: Discipline of Medical Biochemistry, Nelson R Mandela School of Medicine, University of KwaZulu-Natal, Private Bag 7, Congella, 4013, Durban, South Africa

**Keywords:** *Moringa oleifera*, Drumstick tree, Lung cancer, Oxidative stress, Nrf2, Apoptosis

## Abstract

**Background:**

The incidence of lung cancer is expected to increase due to increases in exposure to airborne pollutants and cigarette smoke. *Moringa oleifera* (MO), a medicinal plant found mainly in Asia and South Africa is used in the traditional treatment of various ailments including cancer. This study investigated the antiproliferative effect of MO leaf extract (MOE) in cancerous A549 lung cells.

**Methods:**

A crude aqueous leaf extract was prepared and the cells were treated with 166.7 μg/ml MOE (IC_50_) for 24 h and assayed for oxidative stress (TBARS and Glutathione assays), DNA fragmentation (comet assay) and caspase (3/7 and 9) activity. In addition, the expression of Nrf2, p53, Smac/DIABLO and PARP-1 was determined by Western blotting. The mRNA expression of Nrf2 and p53 was assessed using qPCR.

**Results:**

A significant increase in reactive oxygen species with a concomitant decrease in intracellular glutathione levels (*p* < 0.001) in MOE treated A549 cells was observed. MOE showed a significant reduction in Nrf2 protein expression (1.89-fold, *p* < 0.05) and mRNA expression (1.44-fold). A higher level of DNA fragmentation (*p* < 0.0001) was seen in the MOE treated cells. MOE’s pro-apoptotic action was confirmed by the significant increase in p53 protein expression (1.02-fold, *p* < 0.05), p53 mRNA expression (1.59-fold), caspase-9 (1.28-fold, *p* < 0.05), caspase-3/7 (1.52-fold) activities and an enhanced expression of Smac/DIABLO. MOE also caused the cleavage and activation of PARP-1 into 89 KDa and 24 KDa fragments (*p* < 0.0001).

**Conclusion:**

MOE exerts antiproliferative effects in A549 lung cells by increasing oxidative stress, DNA fragmentation and inducing apoptosis.

## Background

Lung cancer is a leading cause of morbidity and mortality in many countries [1]. Inhalation of airborne pollutants, exposure to toxins present in grain dusts and fungal spores and cigarette smoking causes lung damage and increases the risk of carcinogenesis [2]. South Africa (SA) has the highest human immunodeficiency virus (HIV) infection burden globally and Bello et al. (2011) showed that surviving HIV positive individuals have a high risk of cancer such as lung cancer. Cancer deaths accounted for 63% in developing countries across the world [3]. Cancer is characterised by uncontrolled cell growth as cells proliferate and evade apoptosis [4]. Apoptosis is regulated by caspases through two pathways, viz., death receptor-mediated procaspase-activation pathway (extrinsic pathway) and mitochondrion-mediated procaspase-activation pathway (intrinsic pathway) [4,5]. To maintain cellular homeostasis, these cells follow a process of growth, division and cell death. When this process is affected, it can result in the initiation of cancer.

There are many regulators of apoptosis. The p53 tumor suppressor protein and transcription factor is up-regulated when DNA is damaged by causing G1 arrest and DNA repair; if the repair is unsuccessful then it signals for apoptosis and ultimately cell death [6,7]. During apoptosis cellular proteins are proteolysed by caspases. These proteins also include poly (ADP ribose) polymerase (PARP-1) [8].

Lung cancer still remains incurable and current drug therapies have many side-effects and alternate therapy is actively being sought [9]. If traditional medicine can provide an alternate source for treatment, the number of lung cancer deaths can be reduced. Some traditional medicines possess antiproliferative effects such as *Sutherlandia frutescens,* commonly referred to as cancer bush, is used by traditional healers in SA to treat cancer [10]. *Moringa oleifera* (MO), an indigenous tree to India, is found widely in SA [11]. It belongs to the family Moringaceae and is cultivated for medicinal and industrial purposes [12]. It is commonly referred to as the ‘tree of life’ or Drumstick tree [12,13]. All parts of the MO plant possess medicinal properties, but the leaves have high nutritional value (high levels of vitamins C and A, potassium, proteins, calcium and iron) [14,15]. In addition the leaves possess phytochemicals like carotenoids, alkaloids and flavonoids [11] and is rich in amino acids such as cystine, lysine, methionine and tryptophan [16]. MO is used in traditional treatment of diabetes mellitus, cardiovascular and liver disease.

Phytochemical properties of MO play an important role in its mode of action against diseases [11]. It contains a rich source of rhamnose, glucosinolates and isothiocyanates. A study conducted by Manguro and Lemmen (2007) into the phenolics of MOE had characterised five flavonol glycosides using spectroscopic methods [17]. The anticancer property can be attributed to specific components of MOE such as 4-(α-L-rhamnopyranosyloxy) benzyl glucosinolate, 4-(α-L-rhamnopyranosyloxy) benzyl isothiocyanate, benzyl isothiocyanate and niazimicin. The leaves contain quercetin-3-O-glucoside and kaempferol-3-O-glucoside which plays a role in antioxidant defence as it scavengers for free radicals thus reducing oxidative stress [12]. Thiocarbamates such as niazimicin are found in the leaves and can be used as a chemopreventive agent [18,19]. Studies have suggested that the anticancer and chemopreventive property of MOE can be attributed to niazimicin [20,21].

MO leaf extracts have been shown to disrupt proliferation of cancer cells. In a study on Swiss mice, MO leaf extracts increased glutathione-S-transferase (GST) [12]. The MO leaf extracts induced apoptosis in KB carcinoma cells [22]. Sreelatha and Padma (2011) had shown that the extracts inhibited lipid peroxidation as it scavenged free radicals and reduced oxidative stress [22]. It also protected against oxidative DNA damage. To date there is no study assessing the effects of MO leaf extracts on lung carcinogenesis. The present study investigated the antiproliferative effects of a crude aqueous extract of MO leaves in A549 (human lung carcinoma) cells. It was hypothesised that MO leaf extracts induces cell death as a result of oxidative stress in the cancerous cells.

## Methods

### Materials

MO leaves were collected from the KwaZulu-Natal region (Durban, South Africa) and verified by the KwaZulu-Natal herbarium (Batch no. CT/1/2012, Genus no. 3128). A549 cells were purchased from Highveld Biologicals (Johannesburg, South Africa). Cell culture reagents were purchased from Whitehead Scientific (Johannesburg, South Africa). ECL-LumiGlo® chemiluminescent substrate kit was purchased from Gaithersburg (USA) and western blot reagents were purchased from Bio-Rad (USA). All other reagents were purchased from Merck (South Africa).

### Cell culture

A549 lung cells were cultured (37°C, 5% CO_2_) in 25 cm^3^ culture flasks in complete culture media (CCM) [23] comprising of Eagle’s minimum essential medium supplemented with 10% foetal calf serum, 1% L-glutamine and 1% penicillin-streptomycin-fungizone until confluent [24]. Cell growth was monitored and CCM was changed as necessary. Confluent flasks were trypsinized using 1 ml trypsin. Cell numbers were enumerated using trypan blue.

### Leaf extract

The MO leaf extract (MOE) was prepared by crushing 10 g of air-dried leaves in a pestle and mortar and the subsequent addition of 100 ml de-ionised water [24,25]. The resultant extract was boiled with continuous stirring for 20 min, transferred to 50 ml conical tubes and centrifuged [720 × g, 10 min, room temperature (RT)]. The upper aqueous layer (MOE) was removed, lyophilised and stored at 4°C. MOE stock solution was prepared by dissolving 1 mg of MOE in 1 ml of CCM and filter sterilised [0.22 μM filter (Millipore)].

### Cell viability assay

The cytotoxicity of MOE in A549 cells was determined using the Methyl thiazol tetrazolium (MTT) assay [26]. A549 cells (15,000 cells/well) were seeded into a 96-well microtitre plate. The cells were incubated with varying MOE dilutions (0, 1, 10, 50, 100, 150, 200, 250, 500 μg/ml) in six replicates (300 μl/well) and incubated (37°C, 5% CO_2_) for 24 h. A control of cells incubated with CCM only was used. A CCM/MTT salt solution (5 mg/ml) was added (120 μl/well) and the plate was incubated (37°C, 4 h). Thereafter, supernatants were removed; dimethyl sulphoxide (DMSO) 100 μl/well was added and incubated (1 h). The optical density of the formazan product was measured at 570 nm and reference wavelength of 690 nm using a spectrophotometer (Bio Tek μQuant). The percentage cell viability was determined and a concentration-response curve was plotted using GraphPad Prism V5.0 software relative to the control. This experiment was repeated on two separate occasions before the concentration of half the maximum inhibition (IC_50_) was calculated.

For subsequent assays, A549 cells at inoculation density of 20,000 cells per well were treated (24 h) with the IC_50_ determined on viability assay.

### Lipid peroxidation - quantification of malondialdehyde (MDA)

To investigate MOE generation of reactive oxygen species (ROS), Thiobarbituric acid assay (TBARS) was used. TBARS measures MDA which is the end product of lipid peroxidation and an indicator of oxidative stress [27]. Following treatment, cells lysed in 0.2% H_3_PO_4_ (100 μl) by passing the cell solution through a 25 gauge needle at least 25 times from each sample was transferred to test tubes with the addition of 2% H_3_PO_4_ (200 μl), 7% H_3_PO_4_ (400 μl) and TBA/BHT solution (400 μl). A positive control of MDA and a negative control of CCM were prepared. All samples were adjusted to pH 1.5 and heated (100°C, 15 min). After cooling, butanol (1.5 ml) was added to each test tube, vortexed and allowed to separate into distinct phases. The upper butanol phase (800 μl) was transferred into eppendorfs and centrifuged (17,949 × g, 6 min, RT). 100 μl from each sample was aliquoted into a 96-well microtitre plate in six replicates. The optical density was measured on a spectrophotometer at 532 nm with reference wavelength of 600 nm. The mean optical density for each sample was calculated and divided by the absorption coefficient (156 mM^-1^). The results were expressed in μM.

### Antioxidant potential - quantification of glutathione

Glutathione-Glo™ Assay (Promega) was used according to manufacturer’s guidelines to quantify glutathione (GSH) levels. 50 μl from each sample (MOE treated and untreated control) was added in six replicates to the wells of an opaque polystyrene 96-well microtitre plate. GSH standards (0-50 μM) were prepared from a 5 mM stock diluted in de-ionised water. 50 μl of each GSH standards and 50 μl of the GSH-Glo™ Reagent 2× was added per well and incubated in the dark (30 min, RT). Reconstituted Luciferin Detection Reagent (50 μl) was added per well and incubated (15 min, RT). The luminescence was measured on a Modulus™ microplate luminometer (Turner Biosystems, Sunnyvale, USA). The data was analysed and expressed as relative light units (RLU).

### DNA damage

DNA damage was determined using the Comet assay [28]. Following treatment of cells (20,000 cells/well) in a 6-well plate, supernatants were removed and cells were trypsinized. Three slides per sample were prepared as the first layer of 1% low melting point agarose (LMPA, 37°C), second layer of 25 μl of cells (20, 000) from the samples with 175 μl of 0.5% LMPA (37°C) and third layer of 0.5% LMPA (37°C) covered the slides. After solidification, the slides were then submerged in cold lysing solution [2.5 M NaCl, 100 mM EDTA, 1% Triton X-100, 10 mM Tris (pH 10), 10% DMSO] and incubated (4°C, 1 h). Following incubation the slides were placed in electrophoresis buffer [300 mM NaOH, 1 mM Na_2_EDTA (pH 13)] for 20 min and thereafter subjected to electrophoresis (25 V, 35 min, RT) using Bio-Rad compact power supply. The slides were then washed 3 times with neutralisation buffer [0.4 M Tris (pH 7.4)] for 5 min each. The slides were stained overnight (4°C) with 40 μl ethidium bromide (EtBr) and viewed with a fluorescent microscope (Olympus IXSI inverted microscope with 510-560 nm excitation and 590 nm emission filters). Images of 50 cells and comets were captured per treatment and the comet tail lengths were measured using Soft imaging system (Life Science - ^©^Olympus Soft Imaging Solutions v5) and expressed in μm.

### Caspase-3/7 and 9 activities

Caspase-Glo® 3/7 and Caspase-Glo® 9 Assays (Promega) were used to assess apoptosis. For each assay the same procedure was followed: A549 cells were seeded into an opaque polystyrene 96-well microtitre plate in six replicates. Following treatment, the Caspase-Glo® 3/7 and Caspase-Glo® 9 reagents were prepared according to manufacturer’s guidelines. 100 μl of the reagent was added per well and incubated in the dark (30 min, RT). Following incubation, the luminescence was measured on a Modulus™ microplate luminometer. The data was expressed as RLU and fold change.

### Western blotting

Western Blots were performed to determine the expression of Nrf2, p53, Smac/DIABLO and PARP-1. Briefly, total protein was isolated using Cytobuster™ reagent supplemented with protease inhibitor (Roche, cat. no. 05892791001) and phosphatase inhibitor (Roche, cat. no. 04906837001). The bicinchoninic acid assay (Sigma, Germany) was used to quantify the protein and was standardised to 2.042 mg/ml [29]. The samples were prepared in Laemmli buffer [30], boiled (100°C, 5 min) and electrophoresed (150 V, 1 h) in 7.5% sodium dodecyl sulfate polyacrylamide gels using a Bio-Rad compact power supply. The separated proteins were electro-transferred to nitrocellulose membrane using the Trans-Blot® Turbo Transfer system (Bio-Rad) (20 V, 45 min). The membranes were blocked (1 h) using 3% BSA in Tris-buffered saline containing 0.5% Tween20 (TTBS - NaCl, KCL, Tris, Tween 20, dH_2_O, pH 7.4). Thereafter, the membranes were immune-probed with primary antibody [Nrf2 (ab89443), p53 (ab26), PARP-1 (ab110915), 1:1,000; Smac/DIABLO (ab68352), 1:200] at 4°C overnight. The membranes were then washed 4× with TTBS (10 min each) and incubated with the secondary antibody (ab97046; 1:2,000) at RT for 1 h. The membranes were finally washed 4× with TTBS (10 min each). To correct for loading error and to normalise the expression of the proteins, β-actin was assessed (ab8226; 1:5,000). Horse radish peroxidase (HRP) chemiluminescence detector and enhancer solution was used for the antigen-antibody complex and the signal was detected with the Alliance 2.7 image documentation system (UViTech). The expression of the proteins were analysed with UViBand Advanced Image Analysis software v12.14 (UViTech). The data was expressed as relative band density (RBD) and fold change.

### Quantification of mRNA

To determine p53 and Nrf2 mRNA expression, RNA was first isolated from control and MOE treatment by adding 500 μl Tri reagent (Am9738) as per manufacturer’s guidelines. Thereafter, RNA was quantified (Nanodrop 2000) and standardised to 100 ng/μl. RNA was reverse transcribed by reverse transcriptase into copy DNA (cDNA) using the RT^2^ First Strand Kit (SABiosciences, C-03) as per manufacturer’s instructions. Briefly, a 20 μl reaction was prepared by adding 10 μl genomic DNA elimination mixture (Total RNA, 5× gDNA elimination buffer, H_2_O) to 10 μl of RT cocktail (5× RT buffer 3, primer and external control mix, RT enzyme mix, H_2_O). The reaction was then subjected to 42°C (15 min) and 95°C (5 min) (GeneAmp® PCR System 9700, Applied Biosystems) to obtain cDNA. Quantitative PCR (qPCR) was used to determine mRNA expression using RT^2^ SYBR® Green qPCR Master Mix (SABiosciences). A 25 μl reaction consisting of 12.5 μl IQ™ SYBR® green supermix (cat. no. 170–8880), 8.5 μl nuclease-free water, 2 μl cDNA, and 1 μl sense and anti-sense primer (10 mM, inqaba biotec™, Table 1) were used. The mRNA expression was compared and normalised to a housekeeping gene, GAPDH.

**Table 1:** Primer sequences used in qPCR assay

	**Primer sequence**	
	**Sense Primer**	**Anti-sense Primer**
Nrf2	5′AGTGGATCTGCCAACTACTC 3′	5′CATCTACAAACGGGAATGTCTG 3′
p53	5′CCACCATCCACTACAACTACAT3′	5′CAAACACGGACAGGACCC3′
GAPDH	5′TCCACCACCCTGTTGCTGTA3′	5′ACCACAGTCCATGCCATCAC3′

The reaction was subjected to an initial denaturation (95°C, 10 min). It was followed by 40 cycles of denaturation (95°C, 15 s), annealing (Nrf2: 57°C, 40s; p53: 56°C, 40 s) and extension (72°C, 30 s) (Chromo 4 Real-Time PCR detector, Biorad). The data was analysed using MJ opticon monitor analysis software V3.1, Biorad. The mRNA expression was determined using the Livak method and expressed as fold changes [31].

### Statistical analysis

Statistical analyses were performed using GraphPad Prism v5.0 software (GraphPad Software Inc., La Jolla, USA). The results were expressed as means with standard deviation (SD). The concentration-response-inhibition equation was used to determine IC_50_ for MTT assay. The statistical significances were determined by unpaired *t*-test and a 95% confidence interval. The data were considered statistically significant with a value of *p* < 0.05.

## Results

### Cell viability assay

The MTT assay measures cell viability based on the generation of reducing equivalents in metabolic active cells. The A549 cell viability (%) data is presented in Table 2.

**Table 2:** Viability of A549 cells treated with MOE for 24 h

**Concentration (μg/ml)**	**Mean OD ± SD**	**Cell viability (%)**
0 (Control)	1.469 ± 0.008	100
1	1.177 ± 0.058	80.123
10	1.120 ± 0.132	76.242
50	1.001 ± 0.118	68.108
100	1.201 ± 0.082	81.756
150	1.170 ± 0.110	79.646
200	0.966 ± 0.158	65.725
250	0.922 ± 0.177	62.730
500	0.984 ± 0.350	66.950

*OD* optical density, *SD* standard deviation.

Using GraphPad prism, an IC_50_ value of 166.7 μg/ml was calculated. This concentration of MOE was used in all subsequent assays.

### Assessment of oxidative stress

**Figure 1:**
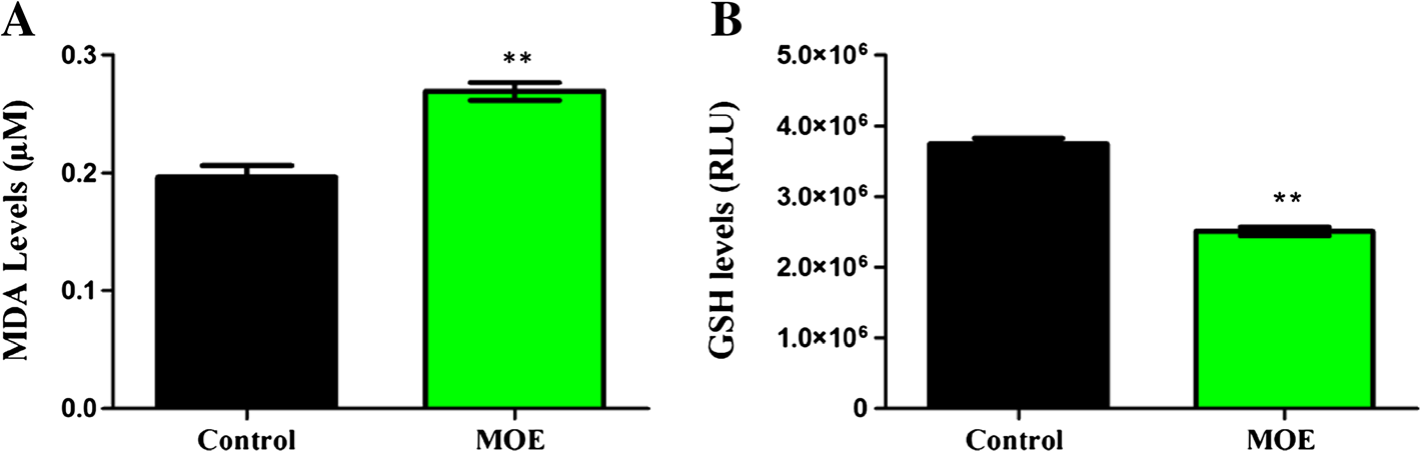
**Oxidative stress induced by MOE on A549 cells.** An increase in MDA levels (lipid peroxidation) **(A)** and decreased intracellular GSH levels **(B)** in MOE treated cells (**p < 0.001).

Reactive oxygen species (ROS) induce oxidative stress. Lipid peroxidation, caused by ROS, was assayed by quantifying MDA presented in Figure 1A.

There was a significant increase in MDA levels in MOE treatment as compared to the untreated cells (0.269 ± 0.013 μM vs 0.197 ± 0.016 μM, *p* < 0.001). GSH levels were significantly decreased in the MOE treatment compared to the control [Figure 1B (2.507 × 10^6^ ± 0.081 × 10^6^ RLU vs 3.751 × 10^6^ ± 0.110 × 10^6^ RLU, *p* < 0.001): Additional file 1].

### DNA damage

**Figure 2:**
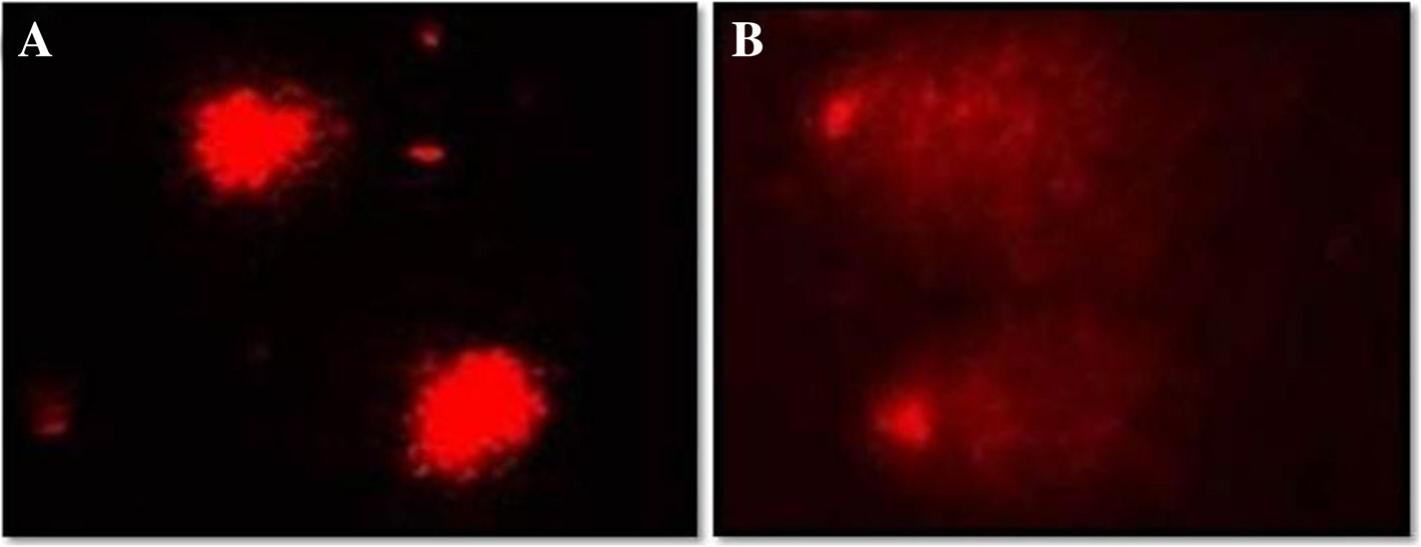
**Comet assay images of control and MOE treatments for 24 h.** DNA damage was higher in cells exposed to MOE **(B)** then control cells **(A)** (100×, ***p < 0.0001).

The comet assay assessed DNA damage and the comet tail lengths were measured in MOE treated and untreated A549 cells (Figure 2).

There was a significant increase in comet tail length in MOE treatment compared to the control (18.52 ± 4.90 μm vs 5.15 ± 1.18 μm, *p* < 0.0001).

### Assessment of caspase-3/7 and 9 activities

Intracellular activity of caspases-3/7 and caspase-9 was measured. Table 3 presents the apoptotic induction in A549 cells.

**Table 3:** Apoptotic markers of A549 cells following treatment for 24 h

	**Mean ± SD (RLU x 10**^**5**^**)**		**Fold change**	***p*-value**
	**Control**	**MOE**		
Caspase-3/7	2.097 ± 0.489	3.196 ± 0.261	1.52	0.107
Caspase-9	12.630 ± 0.020	16.160 ± 0.702	1.28	< 0.05*

*p < 0.05: statistically significant compared to the control, *SD*: standard deviation, *RLU* relative light units.

There was an increase (non-significant) in caspase-3/7 activity and a significant increase in caspase-9 activity in MOE treatment compared to the control (Table 3).

### Western blotting

**Figure 3:**
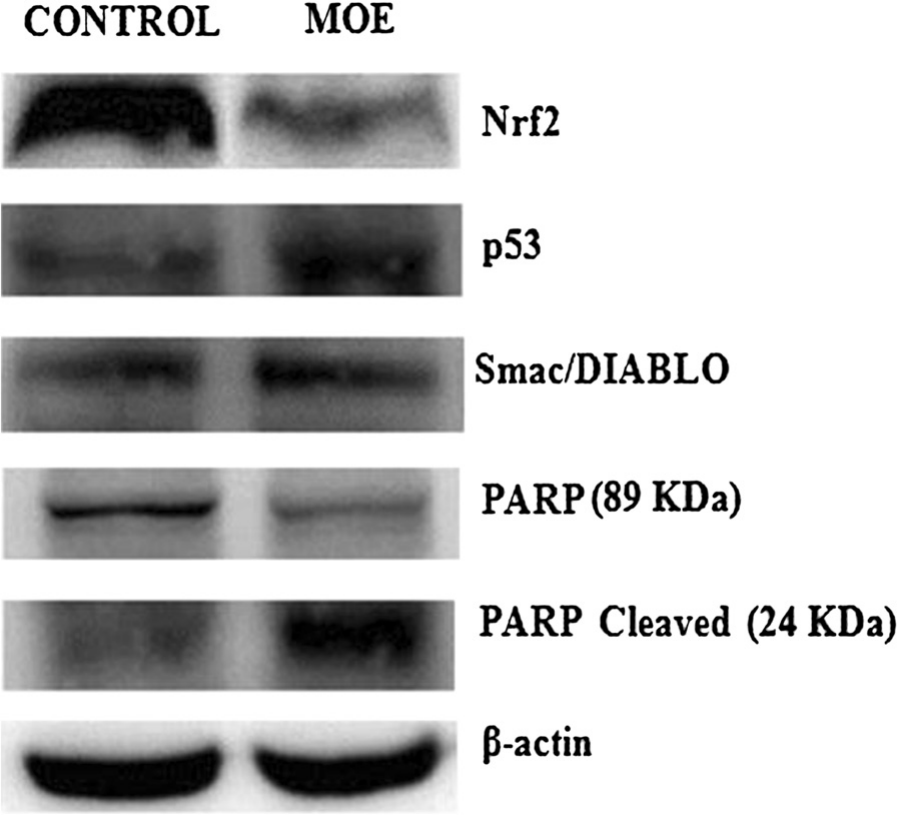
**MOE regulating protein expression in A549 cells.** Differential expression of Nrf2, p53, Smac/DIABLO, PARP-89 KDa and 24 KDa fragment in A549 cells after treatment with MOE for 24 h.

To determine the effect of MOE on protein expression we assessed the levels of Nrf2, p53, Smac/DIABLO and PARP-1 using western blot (Figure 3).

MOE induced a significant 1.89-fold decrease in Nrf2 expression [Figure 3 (0.069 ± 0.007 RBD vs control: 0.129 ± 0.022 RBD, *p* < 0.05)]; a 1.02-fold increase in p53 expression [Figure 3 (0.567 ± 0.002 RBD vs control: 0.558 ± 0.002 RBD, *p* < 0.05)] and a 1.06-fold increase in Smac/DIABLO expression [Figure 3 (1.509 ± 0.055 RBD vs control: 1.425 ± 0.007 RBD, *p* = 0.162)]. During apoptosis, PARP-1 is proteolysed by caspases to an 89 KDa and 24 KDa fragment. There was a significant 1.27-fold decrease in the expression of PARP 89 KDa fragment in the MOE treatment compared to the control [Figure 3 (0.234 ± 0.005 RBD vs 0.297 ± 0.005 RBD, *p* < 0.0001)] and a 1.46-fold increase in the level of PARP 24 KDa fragment [Figure 3 (0.419 ± 0.014 RBD vs 0.286 ± 0.016 RBD, *p* < 0.0001); Additional file 1].

### Quantification of mRNA

**Figure 4:**
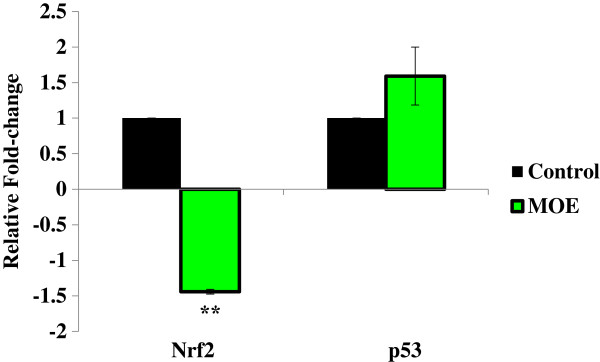
**The effect of MOE on mRNA expression.** MOE regulated the Nrf2 and p53 mRNA expression in A549 cells after treatment for 24 h (**p < 0.001).

The mRNA expression of Nrf2 and p53 in A549 cells was determined using qPCR relative to the control (Figure 4).

The Nrf2 mRNA expression was decreased 1.44 ± 0.03-fold (*p* < 0.001) in MOE treatment (Figure 4). A 1.59 ± 0.41-fold (*p* = 0.168) increase in p53 mRNA expression was observed in MOE treated cells.

## Discussion

MO, a widely consumed traditional plant, is used to treat various ailments such as cancer [13]. Cancer is listed as the fourth leading cause of death in SA [3], with lung cancer expected to increase. This is a first study to show a possible biochemical mechanism of action of MOE on cancerous A549 cells.

Reactive oxygen species are known to induce many diseases [32]. These oxidants damage membrane phospholipids and results in lipid peroxidation [2,27]. This study showed that MOE significantly increased lipid peroxidation as measured by elevated levels of MDA. This lipid peroxidation compromises cell membranes and their function. In addition, the mitochondrial membranes may become dysfunctional and lead to uncoupling of oxidative phosphorylation and increased electron leak from the respiratory chain. These oxidants also react with proteins and DNA in the cell [33].

Hydrogen peroxide (H_2_O_2_) oxidises cysteine in GSH to produce glutathione disulphide (GSSG), thereby decreasing the antioxidant capacity of GSH. GSH levels were significantly decreased in MOE-treated A549 cells with a corresponding significant increase in lipid peroxidation (Figure 1).

The mRNA plays a pivotal role in protein synthesis as it is used as a template and thus translated into protein [34]. The transcription factor nuclear factor-erythroid 2 p45-related factor 2 (Nrf2) is important in antioxidant defence as it protects the cell from oxidative stress. Nrf2 dissociates from Kelch-like epichlorohydrin-associated protein 1 (Keap1) and translocates to the nucleus and binds to the antioxidant-response elements in promoter regions of antioxidant genes thus increasing transcription [35,36]. Nrf2 regulates the synthesis of GSH and MOE reduced mRNA expression by 1.44-fold (Figure 4) [34]. This resulted in a significant decrease in Nrf2 protein expression in A549 cells, (Figure 3) which leads to decreased transcription of important antioxidant genes and increased oxidative damage [37]. The suppression of Nrf2 expression may explain the antiproliferative effect of MOE in this cell line. A consequence is that the endogenous antioxidant GSH is not replenished adequately and will result in increased oxidants and ultimately to cell death.

The increase in oxidative stress is genotoxic to the cell. H_2_O_2_ can react with metal ions such as iron and produce highly reactive hydroxyl radicals that target DNA [22]. ROS-mediated DNA damage can be a therapeutic target in cancer cells as it signals nucleases to cause DNA strand breaks. The MOE induced significant DNA strand breaks and fragmentation in the alveolar epithelial cells (Figure 2). Again this finding shows that MOE possess pro-apoptotic and antiproliferative properties.

To further confirm the pro-apoptotic action of MOE, we investigated its effect on p53 mRNA and protein expression. MOE increased p53 mRNA expression (Figure 4) with a significant increase in the expression of p53 protein in A549 treated cells (Figure 3). It is known that an increase in oxidative stress and DNA damage results in apoptosis [38,39]. DNA damage up-regulates signals for repair and apoptosis. The increased expression of p53 correlates well with the increased DNA damage by MOE. This signals for apoptosis via Bax activation, a pro-apoptotic protein, which causes mitochondrial depolarisation and cytochrome c release from the mitochondria into the cytoplasm. Cytochrome c, together with Apaf-1 and ATP forms an apoptosome resulting in pro-caspase-9 cleavage and activation of caspase-9. MOE significantly increased (1.28-fold) caspase-9 activity, which in turn activates the executioner caspases-3/7 (1.52-fold increase) (Table 3). Caspase-3/7 activity can be inhibited by inhibitor of apoptosis (IAP) proteins [40,41]. The protein, Smac/DIABLO is concurrently released from the mitochondria with cytochrome c and inhibits IAP proteins thus ensuring execution of apoptosis. MOE afforded a slight increase on Smac/DIABLO expression (1.06-fold) [Figure 3 (1.509 ± 0.055 RBD vs control: 1.425 ± 0.007 RBD, *p* = 0.162)] that could contribute to the apoptotic pathway.

In addition PARP-1 cleavage was investigated. During apoptosis, caspases are activated resulting in the cleavage of PARP-1 [6]. PARP-1, a nuclear enzyme, is proteolysed to an 89 KDa C-terminal catalytic fragment and a 24 KDa N-terminal DNA-binding domain fragment [42]. PARP-1 (important in DNA base excision repair) maintains the integrity of the genome [43]. MOE increased caspase-3/7 activity in A549 cells which resulted in cleavage of PARP-1 into 2 fragments [44]. There was a significant (1.46-fold) increase in the expression of the 24 KDa fragment (Figure 3) in MOE treated cells. This increased cleavage of the smaller PARP-1 fragment correlates well with the increased DNA damage by MOE (*p* < 0.0001).

The phytoconstituents of MOE were shown to possess antiproliferative effects on various cell lines [20]. The leaves contain glucosinolates, isothiocyanates, niazimicin, niaziminin and quercetin which attributes to the anticancer effect [11,20,21]. In addition the leaves also contain other thiocarbamate, carbamates and nitrile glycosides [20].

A recent study showed the significance of MO phytochemicals in prostate cancer therapy [21]. Niazimicin and 4-(4’-O-acetyl-α-L-rhamnopyranosyloxy) benzyl isothiocyanate were identified as natural anticancer agents and compared favourably with the recommended chemotherapeutic drug, Estramustine. These phytochemicals enhanced the activity of cellular prostatic acid phosphatase and possessed less toxicity, thus showing potential as a potent and safe natural agent in prostate cancer therapy and drug design [21]. Similarly these active compounds in MOE can act as anticancer agents in lung cancer by inducing cellular apoptosis and subsequent cell death.

An *in vivo* study on the anticancer activity of MOE on B16 F10 melanoma tumors in mice, revealed that treatment at 500 mg/kg-bw could delay tumor growth and increase lifespan [20]. The anticancer activity was attributed to the phytochemicals quercetin, niazimicin and niaziminin. The therapeutic and nutritional use of MOE is safe at doses below 2 g/kg-bw [45]. Similarly the antiproliferative effect of MOE observed in the A549 cancerous cells may be due to the phytochemicals (e.g., isothiocyanates, niazimicin, niaziminin and quercetin) in the plant leaves.

## Conclusion

The MO leaves possess antiproliferative properties as evidenced by an increase in oxidative stress leading to apoptosis of lung cancer cells. The results from the study provide a biochemical mechanism underlying the usage of MOE as a therapeutic agent in lung cancer therapy. It shows a promising complementary and alternative treatment for lung cancer. Furthermore, phytochemical analysis and the effect of MOE on other cancerous cell lines need to be assessed.

## Abbreviations

BSA: Bovine serum albumin; CCM: Complete culture media; cDNA: Copy DNA; DMSO: Dimethyl sulphoxide; EtBr: Ethidium bromide; GST: Glutathione-S-transferase; GSH: Glutathione; GSSG: Glutathione disulphide; HIV: Human immunodeficiency virus; HRP: Horse radish peroxidase; H2O2: Hydrogen peroxide; IAP: Inhibitor of apoptosis; Keap1: Kelch-like epichlorohydrin-associated protein 1; LMPA: Low melting point agarose; MDA: Malondialdehyde; mRNA: Messenger RNA; MO: *Moringa oleifera*; MOE: MO leaf extract; MTT: Methyl thiazol tetrazolium; Nrf2: Nuclear factor-erythroid 2 p45-related factor 2; OD: Optical density; PARP-1: Poly (ADP ribose) polymerase; ROS: Reactive oxygen species; RT: Room temperature; RLU: Relative light units; RBD: Relative band density; SA: South Africa; SD: Standard deviation; TBARS: Thiobarbituric acid assay; TBA/BHT: Thiobarbituric acid (1%)/0.1 mM butylated hydroxytoluene solution; qPCR: Quantitative polymerase chain reaction.

## Competing interests

The authors declare that they have no competing interests.

## Authors’ contributions

CT conceived the study, designed and conducted all laboratory experiments; analysed and interpreted experimental results and prepared the draft manuscript. AP participated in laboratory experiments. AP and AC participated in the study design, data analysis and manuscript preparations. All authors read and approved the final manuscript.

## Pre-publication history

The pre-publication history for this paper can be accessed here:

http://www.biomedcentral.com/1472-6882/13/226/prepub

## Supplementary Material

Additional file 1 **S1.** Table of contents. **S2.** Comet Assay. **S3 to S9.** Western blotting. Click here for file
