# Supplementary material for: The antiproliferative effect of Moringa oleifera crude aqueous leaf extract on cancerous human alveolar epithelial cells
Source: BMC Complement Altern Med. 2013 Sep 16;13:226. doi: 10.1186/1472-6882-13-226 (PMC3852616; doi:10.1186/1472-6882-13-226)
Supplement: Additional file 1 — S1. Table of contents. S2. Comet Assay. S3 to S9. Western blotting. [file 1472-6882-13-226-S1.doc]

**S1**

**The antiproliferative effect of *Moringa oleifera* crude aqueous leaf extract on cancerous human alveolar epithelial cells**

Charlette Tiloke**,** Alisa Phulukdaree and Anil A. Chuturgoon^#^

*^#^Discipline of Medical Biochemistry, School of Laboratory Medicine and Medical Sciences, College of Health Sciences, University of KwaZulu-Natal, Durban, South Africa*

Charlette Tiloke email: [208501101@stu.ukzn.ac.za](mailto:208501101@stu.ukzn.ac.za)

Alisa Phulukdaree email: [204504283@stu.ukzn.ac.za](mailto:204504283@stu.ukzn.ac.za)

Professor Anil A. Chuturgoon - Corresponding author email: [chutur@ukzn.ac.za](mailto:chutur@ukzn.ac.za)

Tel:    +27 31 260 4404
Fax:   +27 31 260 4785

Postal address: Discipline of Medical Biochemistry, Nelson R Mandela School of Medicine, University of KwaZulu-Natal, Private Bag 7, Congella, 4013, Durban, South Africa

**Table of contents**

1) Comet Assay S2

2) Western blotting S3 – S9

**S2**

**Figure 1** DNA damage was assessed using the Comet assay and comet tails length were measured and compared between the control and treatment groups. MOE significantly increased comet tail length as compared to the untreated control (18.52 ± 4.90µm vs 5.15 ± 1.18µm, *p* < 0.0001).

**S3**


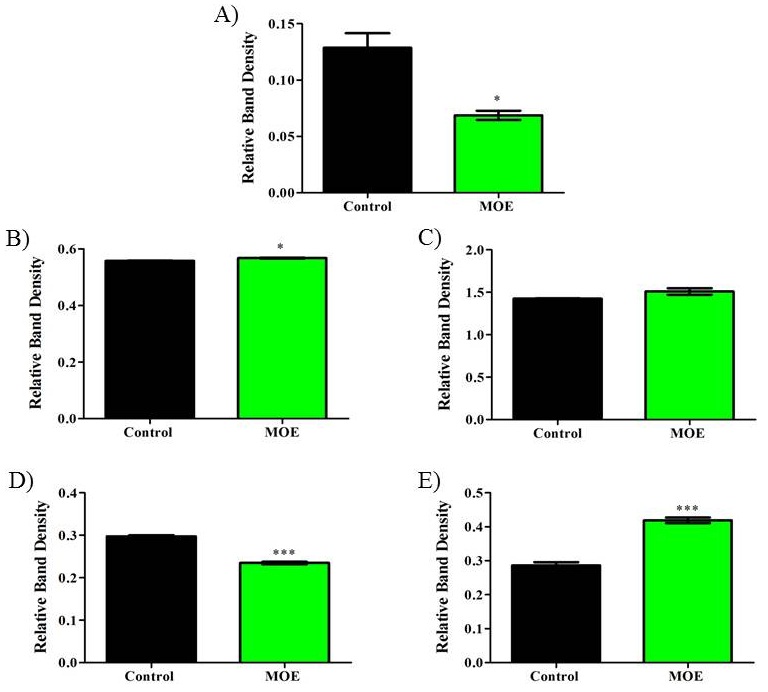


**Figure 2** Western blot was used to determine the expression of proteins in relative band density (RBD) of Nrf2 **(A)**, p53 **(B)**, Smac/DIABLO **(C)**, PARP-89 KDa **(D)** and 24 KDa **(E)** fragment in A549 cells after treatment with MOE. The expression of Nrf2 in MOE treated cells were significantly decreased compared to the untreated control [Figure 2A (0.069 ± 0.007 RBD vs 0.129 ± 0.022 RBD, *p* < 0.05). There was a significant increase in p53 expression [Figure 2B (0.567 ± 0.002 RBD vs control: 0.558 ± 0.002 RBD, *p* < 0.05)] and Smac/DIABLO expression [Figure 2C (1.509 ± 0.055 RBD vs control: 1.425 ± 0.007 RBD, *p* = 0.162)]. MOE caused PARP cleavage into an 89 KDa and 24 KDa fragment with a

**S4**

significant decrease in PARP 89 KDa fragment expression compared to the control [Figure 2D (0.234 ± 0.005 RBD vs 0.297 ± 0.005 RBD, *p* < 0.0001)]. The PARP 24 KDa fragment was significantly increased [Figure 2E (0.419 ± 0.014 RBD vs 0.286 ± 0.016 RBD, *p* < 0.0001)].

**S5**

**Data analysis for western blot**

1) Nrf2

**Table 1 The normalisation of the expression of Nrf2 in the control**

| **Treatments** | **RBD 1** | **RBD 2** | **RBD 3** |
| --- | --- | --- | --- |
| Control | 23118944 | 31714632 | 24647880 |
| β-actin | 205612604 | 205612604 | 205612604 |
|  | 0.112 | 0.154 | 0.120 |

**Table 2 The normalisation of the expression of Nrf2 in the MOE treatment**

| **Treatments** | **RBD 1** | **RBD 2** | **RBD 3** |
| --- | --- | --- | --- |
| MOE | 16219257 | 17313580 | 13970162 |
| β-actin | 230473898 | 230473898 | 230473898 |
|  | 0.070 | 0.075 | 0.061 |

**Table 3 The expression of Nrf2 in A549 cells treated with MOE for 24h**

| **Treatments** | **RBD 1** | **RBD 2** | **RBD 3** | **Mean RBD** | **SD** | **Fold change** |
| --- | --- | --- | --- | --- | --- | --- |
| Control | 0.112 | 0.154 | 0.120 | 0.129 | 0.022 | 1 |
| MOE | 0.070 | 0.075 | 0.061 | 0.069 | 0.007 | 1.89 |

**S6**

2) p53

**Table 4 The normalisation of p53 expression in the control**

| **Treatments** | **RBD 1** | **RBD 2** | **RBD 3** |
| --- | --- | --- | --- |
| Control | 8609 | 8566 | 8636 |
| β-actin | 15394 | 15394 | 15394 |
|  | 0.559 | 0.556 | 0.561 |

**Table 5** The normalisation of p53 expression in the MOE treatment

| **Treatments** | **RBD 1** | **RBD 2** | **RBD 3** |
| --- | --- | --- | --- |
| MOE | 9661 | 9606 | 9648 |
| β-actin | 16986 | 16986 | 16986 |
|  | 0.569 | 0.566 | 0.568 |

**Table 6 The expression of p53 in A549 cells treated with MOE for 24h**

| **Treatments** | **RBD 1** | **RBD 2** | **RBD 3** | **Mean RBD** | **SD** | **Fold change** | |
| --- | --- | --- | --- | --- | --- | --- | --- |
| Control | 0.559 | 0.556 | 0.561 | 0.558 | 0.002 | | 1 |
| MOE | 0.569 | 0.566 | 0.568 | 0.567 | 0.002 | | 1.02 |

**S7**

3) Smac/DIABLO

**Table 7 The normalisation of the expression of Smac/DIABLO in the control**

| **Treatments** | **RBD 1** | **RBD 2** | **RBD 3** |
| --- | --- | --- | --- |
| Control | 22059 | 21905 | 21969 |
| β-actin | 15428 | 15428 | 15428 |
|  | 1.430 | 1.420 | 1.424 |

**Table 8 The normalisation of the expression of Smac/DIABLO** in the MOE treatment

| **Treatments** | **RBD 1** | **RBD 2** | **RBD 3** |
| --- | --- | --- | --- |
| MOE | 22551 | 23743 | 22266 |
| β-actin | 15335 | 15335 | 15335 |
|  | 1.471 | 1.548 | 1.509 |

**Table 9 Smac/DIABLO expression in A549 cells treated with MOE for 24h**

| **Treatments** | **RBD 1** | **RBD 2** | **RBD 3** | **Mean RBD** | **SD** | **Fold change** | |
| --- | --- | --- | --- | --- | --- | --- | --- |
| Control | 1.430 | 1.420 | 1.424 | 1.425 | 0.007 | | 1 |
| MOE | 1.471 | 1.548 | 1.509 | 1.509 | 0.055 | | 1.06 |

**S8**

4) *PARP 89KDa

**Table 10 The normalisation of PARP 89KDa expression** in the control

| **Treatments** | **RBD 1** | **RBD 2** | **RBD 3** |
| --- | --- | --- | --- |
| Control | 6521 | 6369 | 6299 |
| β-actin | 21520 | 21520 | 21520 |
|  | 0.303 | 0.296 | 0.293 |

**Table 11 The normalisation of PARP 89 KDa expression** in the MOE treatment

| **Treatments** | **RBD 1** | **RBD 2** | **RBD 3** |
| --- | --- | --- | --- |
| MOE | 4495 | 4644 | 4662 |
| β-actin | 19623 | 19623 | 19623 |
|  | 0.229 | 0.237 | 0.238 |

**Table 12 The expression of PARP 89 KDa in A549 cells treated with MOE for 24h**

| **Treatments** | **RBD 1** | **RBD 2** | **RBD 3** | **Mean RBD** | **SD** | **Fold change** | |
| --- | --- | --- | --- | --- | --- | --- | --- |
| Control | 0.303 | 0.296 | 0.293 | 0.297 | 0.005 | | 1 |
| MOE | 0.229 | 0.237 | 0.238 | 0.234 | 0.005 | | 1.27 |

**S9**

* PARP 24KDa

**Table 13 The normalisation of the expression of** PARP 24 KDa in the control

| **Treatments** | **RBD 1** | **RBD 2** | **RBD 3** |
| --- | --- | --- | --- |
| Control | 6559 | 5977 | 5941 |
| β-actin | 21520 | 21520 | 21520 |
|  | 0.305 | 0.278 | 0.276 |

**Table 14 The normalisation of the expression of** PARP 24 KDa in the MOE treatment

| Treatments | RBD 1 | RBD 2 | RBD 3 |
| --- | --- | --- | --- |
| MOE | 8452 | 8291 | 7913 |
| β-actin | 19623 | 19623 | 19623 |
|  | 0.431 | 0.423 | 0.403 |

**Table 15 PARP 24 KDa expression in A549 cells treated with MOE for 24h**

| **Treatments** | **RBD 1** | **RBD 2** | **RBD 3** | **Mean RBD** | **SD** | **Fold change** |
| --- | --- | --- | --- | --- | --- | --- |
| Control | 0.305 | 0.278 | 0.276 | 0.286 | 0.016 | 1 |
| MOE | 0.431 | 0.423 | 0.403 | 0.419 | 0.014 | 1.46 |
